# Primary cutaneous follicle center B-cell lymphoma, spindle cell type, arising in background localized lichen myxedematosus: A potential novel association

**DOI:** 10.1016/j.jdcr.2026.04.016

**Published:** 2026-04-16

**Authors:** William Moody, Eric Mou, John Selby, Vincent Liu

**Affiliations:** a University of Iowa Carver College of Medicine, Iowa City, Iowa; b Division of Hematology, Oncology, and Blood & Marrow Transplant, University of Iowa Hospital and Clinics, Iowa City, Iowa; c Department of Dermatology, University of Iowa Hospitals and Clinics, Iowa City, Iowa; d Department of Pathology, University of Iowa Hospitals and Clinics, Iowa City, Iowa

**Keywords:** cutaneous B-cell lymphoma, follicle center B-cell lymphoma, lichen myxedematosus, MGUS, monoclonal gammopathy, multiple myeloma, paraneoplastic, scleromyxedema, spindle cell B-cell lymphoma

## Introduction

Lichen myxedematosus (LM) occupies one end of a spectrum of skin conditions characterized by mucin deposition and the proliferation of fibroblasts, manifesting as papules, plaques, and nodules.^1^ While LM presents locally without systemic involvement, on the other end of the continuum lies scleromyxedema, featuring diffuse skin and systemic involvement with associated monoclonal gammopathy.^2^

LM is typically a benign condition, but rarely, secondary malignancies, including lymphomas, have been reported to concurrently occur.^3^ Herein is described a patient with localized LM without monoclonal gammopathy, who subsequently developed primary cutaneous B-cell lymphoma (CBCL), specifically follicle center B-cell lymphoma, spindle cell type, the first such case reported in the literature to our knowledge (Appendix, available via Mendeley at https://data.mendeley.com/datasets/y245ycdcft/1).

## Case report

**Fig 1:**
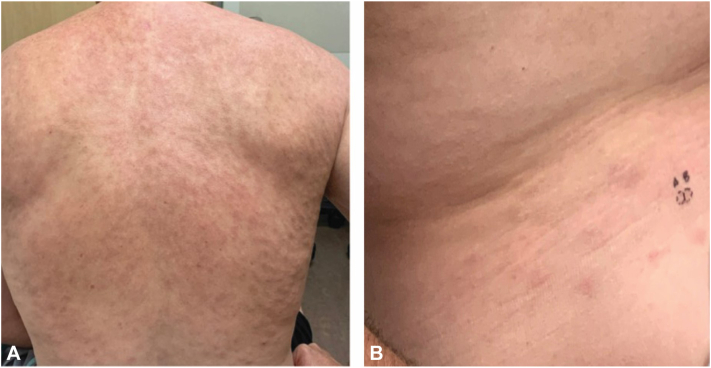
Localized lichen myxedematosus. **(A)** Diffuse erythematous-brown indurated papulonodules on the back at initial consultation. **(B)** Discrete, atrophic macules on the anterior hip 5 months later.

A 53-year-old male with no pertinent medical history presented with erythematous brown, mildly pruritic small papulonodules over the chest, back, neck, arms, and posterior scalp for the previous 2 months, unresponsive to prednisone, prompting evaluation at our institution (Fig 1, *A*).

**Fig 2:**
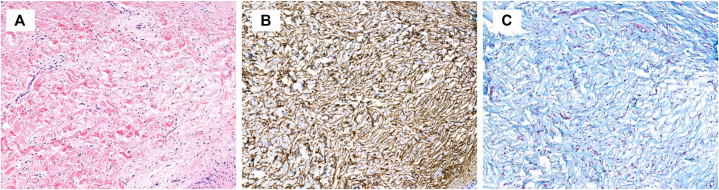
Localized lichen myxedematosus. Skin punch biopsy from the right anterior hip. **(A)** Bland spindle cell proliferation set in background fibrotic and mucinous stroma [H&E; 200×]. **(B)** Spindle cells stain positively for CD34 immunohistochemical stain [CD34; 200×]. **(C)** Interstitial dermal mucin highlighted on Alcian blue stain [Alcian blue; 200×].

Five months later, atrophic macules developed on his anterior hips with a “cigarette paper” appearance (Fig 1, *B*). Skin punch biopsy revealed increased scattered interstitial spindle cells (Fig 2, *A*) with strong diffuse CD34 expression (Fig 2, *B*). The dermis was fibrotic, and increased interstitial mucin was identified (Fig 2, *C*), leading to a diagnosis of localized lichen myxedematosus. Further diagnostic tests, including thyroid-stimulating hormone, as well as serum and urine immunofixation electrophoresis, were unremarkable.

**Fig 3:**
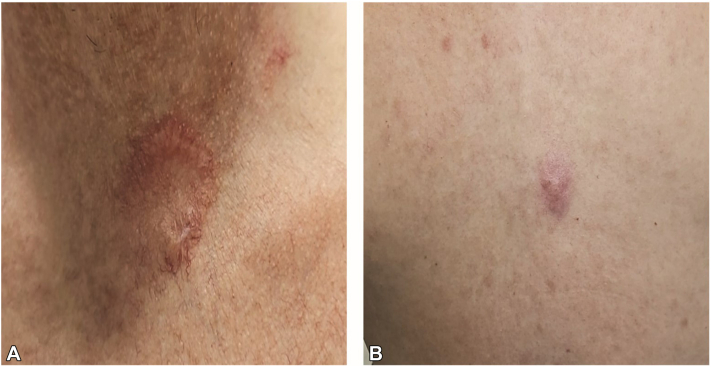
Cutaneous follicle center B-cell lymphoma. **(A)** Firm, pink, arcuate nodular plaque with fine telangiectasias on the left medial clavicle. **(B)** Pink to red patch with centrally located multilobular nodularity on the mid-paraspinal back.

For the next ∼23 m, his skin disease demonstrated marginal improvement following multiple treatments, including prednisone, hydroxychloroquine, UVA-1 phototherapy, methotrexate, isotretinoin, and mycophenolate. Following a 22-m drug holiday, the patient returned to clinic with a firm, pink, arcuate nodule with fine telangiectasias on the left medial clavicle (Fig 3, *A*), a nontender pink to red patch with centrally located multilobular nodularity on the mid-paraspinal back (Fig 3, *B*), and scattered atrophic indentations on the bilateral upper extremities. No lymphadenopathy was appreciated on exam.

**Fig. 4:**
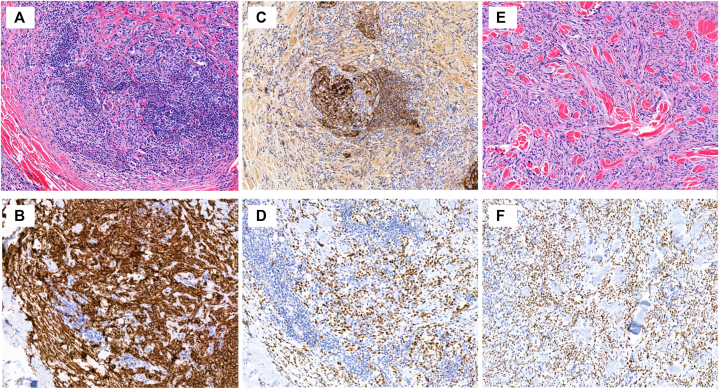
Follicle center B-cell lymphoma. Skin punch biopsy from the left medial clavicle. **(A)** Disrupted germinal center architecture featuring intermediate-sized lymphocytes with irregular nuclear contours [H&E; 200×]. **(B)** Neoplastic cells stain positively for CD20 by immunohistochemistry [CD20; 200×]. **(C)** Follicular dendritic cell meshwork highlighted by CD21 immunohistochemistry [CD21; 200×]. **(D)** Neoplastic cells express BCL6 and infiltrate beyond the confines of the lymphoid follicle [BCL6; 200×]. **(E)** Neoplastic B-lymphocytes showing spindle cell cytomorphology associated with mucinous stroma [H&E; 200×]. **(F)** Spindle cells expressed both CD20 and BCL6, confirming their identity as members of the follicle center lymphoma population [BCL6; 200×].

Skin biopsy of the left medial clavicle lesion showed a nodular and diffuse dermal atypical lymphoid infiltrate manifesting disrupted germinal center architecture featuring intermediate sized lymphocytes with irregular nuclear contours, without appreciable tingible body macrophages or plasma cells (Fig 4, *A*). Neoplastic cells expressed CD20 (Fig 4, *B*), PAX5, and BCL6, but not CD10 or MUM1. Some neoplastic cells expressed BCL2 CD21 highlighted follicular dendritic meshworks in the nodular areas (Fig 4, *C*), with the BCL6-positive neoplastic cellular population spilling out beyond lymphoid follicular architecture. CD3 identified background small reactive T lymphocytes. Furthermore, in extensive regions, tumor cells assumed spindle cell cytomorphology, compatible with elongated centrocytes exhibiting pleomorphic nuclei, interweaving between collagen bundles, and associated with myxoid stroma (Fig 4, *F*). The integrated histomorphologic and immunophenotypic profile was consistent with follicle center B-cell lymphoma, spindle cell type.

Following an unrevealing PET/CT scan, the patient was diagnosed with primary cutaneous follicle center B-cell lymphoma, stage T1aN0M0, involving 1% to 2% of body surface area. He deferred skin-directed treatment, and no systemic therapy was indicated.

## Discussion

We present an unusual case of primary cutaneous follicle center B-cell lymphoma, spindle cell type, developing in the setting of localized LM, a phenomenon rarely, if ever, previously reported in the literature. This combination of individually rare conditions is remarkable not only for its unique co-occurrence but also for its potential in expanding the paraneoplastic associations for localized LM.

Scleromyxedema is recognized as a paraneoplastic dermatosis with a well-established association with monoclonal gammopathy, classically IgG, with lambda light chain restriction. Bone marrow plasmacytosis is occasionally evident, though less than 10% of patients will develop symptomatic myeloma.^1^ Visceral malignancies have also been rarely associated with scleromyxedema.^4^ Highlighting the paraneoplastic connection, there are multiple reports of improvement of scleromyxedema following chemotherapy targeted at the underlying malignancy and further examples of skin lesions regressing following surgical resection of a coexisting malignancy. At the same time, no definitive association between serum paraproteinemia and the disease course of scleromyxedema has been established. However, it has been hypothesized that the 2 conditions may be indirectly linked, perhaps via immune dysregulation or a nonparaprotein circulating factor.^5^

In contrast, LM is a diagnostic entity generally reserved for patients with skin-limited disease without concurrent paraproteinemia.^1^^,^^2^ While there is no known direct connection between atypical B lymphocytes, mucin deposition, and fibroblast proliferation, prior reports have documented LM with co-existing lymphoid malignancies, including pulmonary MALT lymphoma and mycosis fungoides arising in a fibromucinous background.^3^^,^^6^ The pathogenesis of LM is unknown, but the most widely accepted hypothesis centers on circulating cytokines such as IL-1, TNF, and TGF-β, increasing acid glycosaminoglycan synthesis and fibroblast proliferation.^1^ Potentially, localized inflammation and increased TGF-β production by fibroblasts could allow aberrant B-cells to modulate their microenvironment via immune suppression and stromal reprogramming, resulting in a protumoral niche. Bi-directional cross-talk between cells of the microenvironment can promote immune cell suppression and survival of malignant B-cells.^7^ Additionally, CD34+ dermal dendrocytes are known to produce acidic glycosaminoglycans in conjunction with neoplastic conditions, such as dermatofibrosarcoma protuberans.^8^ In our case, it is conceivable that the localized LM background provided an environment that promoted the evolution and progression of CBCL. As a tantalizing overlap between LM and CBCL, mucinous stroma has been associated with primary cutaneous spindle cell B-cell follicle center lymphoma.^9^^,^^10^

Our patient documents the rare development of primary cutaneous follicle center B-cell lymphoma spindle cell type, in the background of localized LM without paraproteinemia, alerting clinicians to the possibility of this unusual co-occurrence. Furthermore, this report compels reconsideration of the traditional conceptualization of the spectrum of skin-limited LM to paraproteinemia-associated scleromyxedema and offers localized LM as a potentially paraneoplastic condition associated with CBCL.

## Conflicts of interest

None disclosed.
